# Peri-implantitis at implants with different diameters: a pilot study in dogs

**DOI:** 10.1186/s40729-019-0177-3

**Published:** 2019-07-01

**Authors:** Fabrizio Morelli, Karol Alí Apaza Alccayhuaman, Paolo Viganò, Franco Bengazi, Joaquin Urbizo, Gianfranco Cesaretti, Daniele Botticelli

**Affiliations:** 1 Faculty of Dentistry, University of Medical Science, La Habana, Cuba; 2 ARDEC Academy, Ariminum Odontologica SRL, Viale Pascoli 67, 47923 Rimini, Italy

**Keywords:** Animal experimentation, Decontamination, Dental implant, Oral surgical procedures, Peri-implantitis

## Abstract

**Aim:**

To evaluate the progression of an induced peri-implantitis at implants with different diameters and the outcome of a corrective surgical debridement.

**Methods:**

Three months after the extraction of the mandibular premolars and first molars in six dogs, non-submerged narrow implants (3.3 mm in diameter) or standard implants (3.8 mm and 4.1 mm) were installed in the molar regions, bilaterally. After 3 months, peri-implantitis lesions were induced with ligatures and plaque accumulation for 3 months. Plaque accumulation was allowed for a further month after ligatures removal. A surgical mechanical decontamination of the surfaces was subsequently performed using gauzes soaked in saline and irrigation. Five months after, biopsies were retrieved and histological slides prepared. X-rays were taken at treatment and 5 months after.

**Results:**

Fourth months after peri-implantitis induction, 2.2 ± 1.0 mm at the standard implants and 3.2 ± 0.4 mm at the narrow implants were observed. Five months after treatment, a mean gain of marginal bone of 0.5 ± 0.6 mm was obtained at the standard implants and of 0.9 ± 0.4 at the narrow implants (*p* = 0.249). The vertical and horizontal defects were found partially closed. At the histological analysis, the coronal level of osseointegration after 5 months of healing was at 2.1 ± 0.8 mm at the standard implants, and 2.8 ± 0.3 mm at narrow implants (*p* = 0.116).

**Conclusions:**

In conclusion, the narrow implants showed a tendency of a faster progression of the induced peri-implantitis compared to standard implants. The implant diameter did not influence the outcome of a surgical treatment of an induced peri-implantitis.

## Introduction

Peri-implantitis is a quite recurrent disease that has been reported to have a prevalence of more than 12% of the implants installed [1]. The diagnosis of peri-implantitis requires the presence of both bleeding on probing and progressive bone loss [2, 3]. These lesions should be treated as earlier as possible to avoid the progression of the disease [4]. A systematic review on the non-surgical treatment of peri-implant disease reported effective result on mucositis. However, modest or non-predictable outcomes were reported for the treatment of peri-implantitis [5].

The surgical treatment of peri-implantitis, provided that a regular supportive care is subsequently applied, may yield improvement of the clinical parameters and marginal bone stability over time [6].

The results of the surgical treatment may also depend on some parameters, such as the surface characteristics [7, 8]. In a retrospective study, the files of 50 patients who underwent surgical treatment for peri-implant disease were analyzed [7]. It was shown that the outcome was positive in the long-term and that it yielded better results at non-modified compared to modified surfaces. In a randomized controlled trial [8], 100 patients were included for surgical treatment of peri-implantitis sites. Systemic antibiotics and/or antiseptic agents were added. The results were compared to control sites that received only the surgical treatment. It was concluded that all treatments were successful and that the systemic antibiotics did not add beneficial effect. Moreover, implants with a non-modified surface yielded better results [8].

The influence of the surface characteristics on peri-implantitis progression was also described in experimental studies [9–12]. In one of these experiments [9], implants with turned or modified surfaces were installed in the mandible of dogs. After having induced peri-implantitis, the ligatures were removed and no plaque control was established for a further 26 weeks. During the period after ligatures removal, larger marginal bone loss and inflammatory infiltrate were observed at the non-modified surface compared to the turned surfaces. Differences in the outcomes were also disclosed when different modified surfaces were compared when a plaque control program was either established [10] or not performed at all [11, 12]. It was also shown that in the absence of an accurate plaque control, inflammatory infiltration and bone resorption were detected independently from the characteristics of the surface. However, the loss of bone was larger at some of the modified surfaces used compared to others.

The outcomes of implants with different diameters have been also evaluated. Similar results in terms of marginal bone level, implant survival, success rate, and clinical performances were found for implants with narrow (3.3 mm) and standard (4.1 mm) diameter, both used in the posterior [13] or in the anterior [14] regions of the mouth. However, the impact of the diameter of the implant on peri-implantitis progression and treatment outcomes needs to be further investigated. Hence, the aim of the present study was to evaluate the progression of an induced peri-implantitis at implants with different diameters and the outcome of a corrective surgical debridement.

The hypothesis was that the diameter of the implants might influence the progression of the disease and the outcome of a corrective surgical debridement.

## Material and methods

Before starting the experiment, the protocol was approved by the Ethical Committee of the University of Medical Science, Faculty of Dentistry, Havana, Cuba (N° 05/2011, 10 February 2011). The ARRIVE checklist (Research Reporting of In Vivo Experiment) was followed. The local rules for animal experiments were rigorously adopted.

### Sample

Six Beagle dogs of 1–2 years of age and about 9–10 kg of weight grown at CENPALAB (Centro Nacional para la Producción de Animales de Laboratorio, Cuba) were included in the experiment. The experiment was performed at the University of Medical Sciences and maintained in couples in kennels on concrete runs located in the facilities of the same university. The animals received moistened balanced dog’s food with free access to the water.

According to the Three R requirements [15], a split-mouth design was adopted to reduce the number of animals. Due to the pre-clinical nature of the present study, in the absence of sound data on the influence of implant dimensions on the outcome of peri-implant treatment, a low number of animals was used, considered however sufficient to describe the healing with acceptable approximation.

### Surgical procedures

**Fig. 1:**
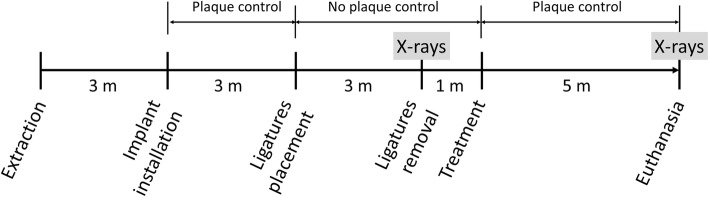
Timeline of the experiment. m = month

The timeline of the experiment is reported in Fig. 1. The surgical procedures were described in a previously published article in which the premolar region of the mandible was used [16]. Briefly, atropine 0.02 mg/kg (Mayne Pharma, Naples, Italy), metedomidine 0.04 mg/kg (Medetor®,Virbac, Glattbrugg, Switzerland), and ketamine-50 5 mg/kg (Laboratorios Liorad, La Habana, Cuba) were injected and the anesthesia was subsequently maintained with Isoflurane-Vet® 2.5% (Merial, Merial, Toulouse, France) mixed to O_2_ at 95%. Anesthesia was also injected in the regions of the experiment. A pharmacologic treatment was also added during the surgery using analgesic (tramadol® 2 mg/kg, Altadol®, Formevet, Milan, Italy) and antibiotics (10 mg/kg, Convenia®, Pfizer, USA).

Three months after the extraction of all mandibular premolars and first molars, implants (Premium®, Sweden & Martina, Due Carrare, PD, Italy) with the same surface [17] (ZirTi, Sweden and Martina, Due Carrare; PD, Italy) and geometry were used. Two control implants, with a standard diameter of 3.8 mm and 4.25 mm respectively, were installed in the distal aspect of the mandible while, in the contralateral side, two “narrow” test implants, 3.3 mm in diameter, were installed. The implants had a 2.2-mm-high polished neck and were placed with the coronal margin of the rough surface at the level of the buccal bony crest. Healing screws were placed on the implants and the flaps were closed with resorbable sutures to allow a non-submerged healing.

Three months after, ligatures were applied deep in the sulci around the four implants aiming to induce peri-implantitis. The ligatures were replaced after 6 weeks and removed after 3 months from the first placement. Plaque accumulation was allowed during the ligatures period and for 1 month more after ligatures removal.

### Corrective surgical debridement

**Fig. 2:**
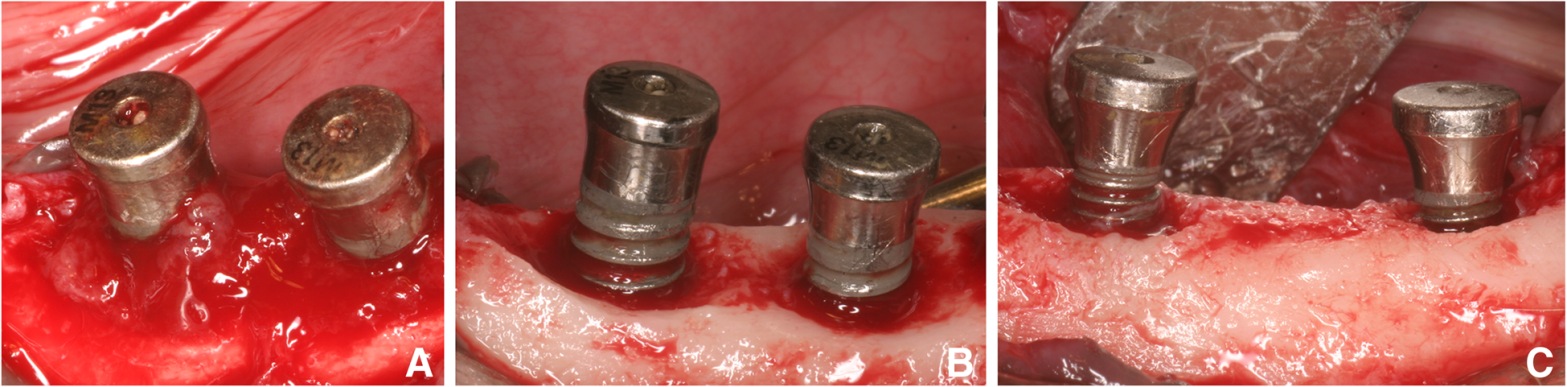
Clinical views. **a** Standard implants with granulation tissue in the marginal sites. **b** Standard implants after treatment. **c** Narrow diameter implant sites at the treatment

After that, a surgical treatment was performed to all sites. The flaps were opened (Fig. 2a), granulation tissue, and calculi were removed with a curette and the surfaces of the implants were cleaned with gauzes soaked in saline, followed by irrigation with saline (Fig. 2b, c). The treatment was repeated 10 times, and clinical measurements were carried out. The flaps were sutured to allow a non-submerged healing.

### Maintenance

Antibiotics (20 mg/kg, Convenia®, Pfizer, NY, USA) and analgesic (2 mg/kg tramadol Altadol®, Formevet, Milan, Italy) were administered every day for 5 days. Daily inspections of the wounds were carried out during the first week after each surgery. Moreover, care of the wounds and a cleaning of the exposed parts of the implants were performed after implants installation and peri-implantitis treatment by veterinaries and veterinary technicians not involved in the experiment.

### Euthanasia

The euthanasia was carried out 5 months after the peri-implantitis treatment. The animals were anesthetized, and the heart was arrested using 25 meq of potassium chloride i.v. (Aica, Havana, Cuba). A fixative (4% formaldehyde solution) was injected through the carotid arteries, and the mandible was removed.

### Histological preparation and evaluation

Block sections were obtained, fixed in formaldehyde, dehydrated, and then included in resin. Ground sections were obtained in a buccal-lingual plane and stained with Stevenel’s blue and alizarin red. (See Viganò et al. 2018 for more information) [16].

### Histological assessments

The histological assessments were performed using an Eclipse Ci microscope (Nikon Corporation, Tokyo, Japan) and the software NIS-Elements D (v 4.0, Laboratory Imaging, Nikon Corporation, Tokyo, Japan). As landmarks were used the implant shoulder (IS), the top of the bone crest (C), the most coronal level of osseointegration (B), and the coronal margin of the rough surface (M). The distance between IS and C (IS-C) and between IS and B (IS-B) were measured. The distances M-B and M-C were calculated subtracting 2.2 mm from IS-B and IS-C, respectively.

### Clinical bone level

Measurement of the level of the buccal and lingual bone crests at the time of peri-implantitis treatment were performed using an UNC 15 probe (Hu-Friedy, Chicago, IL, USA). Due to the larger dimension of the tip of the probe compared to that of the defects, no evaluations of the extension of the loss of osseointegration were performed.

### Radiographic measurements

Digital X-rays were taken during the treatment of the peri-implantitis and after the euthanasia. The software ImageJ (Wayne Rasband, National Institute of Health, Bethesda, MD, USA) was used for measurements and the length of implants was used for calibration.

The same landmarks used in the histological evaluation were applied. Besides M-B and M-C, the horizontal (H-GAP; distance between the implant surface and the bone crest), and vertical defects (V-defect, calculated as the difference between IS-B and IS-C) were assessed.

### Randomization and allocation concealment

One author (DB) not involved in the surgical procedure performed the randomization electronically (randomization.com) to decide the location where to install standard (control) or narrow (test) implants.

The six animals were randomized into one block, and the results were maintained confidentially in sealed opaque envelopes until the implant bed preparation at the surgical session. An expert surgeon performed all surgeries (PV). The expert assessor (KAAA) of the histological slides and of the X-rays was not informed about the aim of the study, even though the different sizes of the implants were recognizable. All histological and radiographic measurements were repeated twice and mean values were used. The intra-class correlation coefficient was > 0.9 for both radiographic and histological assessments.

### Data analysis

The difference (Δ) between the radiographic measurements performed at the mesial/distal aspects at the time of treatment and after 5 months of healing was calculated for the coronal level of osseointegration (ΔM-B), the bone crest height (ΔM-C), the vertical defects (ΔV-defect), and the horizontal gap (ΔGAP). Mean values were obtained between the two standard (control) and the two narrow (test) implants, and the animals were used as units for statistical analysis.

The main variable was the difference in the radiographic level of the most coronal contact of bone to the implant (ΔM-B). The differences in the radiographic assessment of bone crest height (ΔM-C), vertical defect (ΔV-defect), and horizontal gap (ΔGAP) were considered as secondary variables. Histological assessments were used as descriptive data.

The Wilcoxon signed rank test was used for statistical analyses (SPSS v 19, IBM Inc., Chicago, IL, USA). The level of significance was set at α = 0.05.

## Results

The healing period was uneventful and all dogs were healthy at the time of the euthanasia. One 3.3 mm implant was lost before treatment, and the corresponding 4.25 mm was lost after treatment. Mean values obtained between the two standard and the two narrow implants yielded an *n* = 6.

At the time of treatment, all sites presented bleeding on probing. At the standard implants, the bone crest was located apically to the coronal margin of the rough surface (M) 2.2 ± 1.0 mm and 1.6 ± 1.0 mm at the buccal and lingual aspects, respectively. At the narrow implants, the respective levels of the bone crest were 2.7 ± 0.6 mm and 1.6 ± 1.2 mm.

**Fig. 3:**
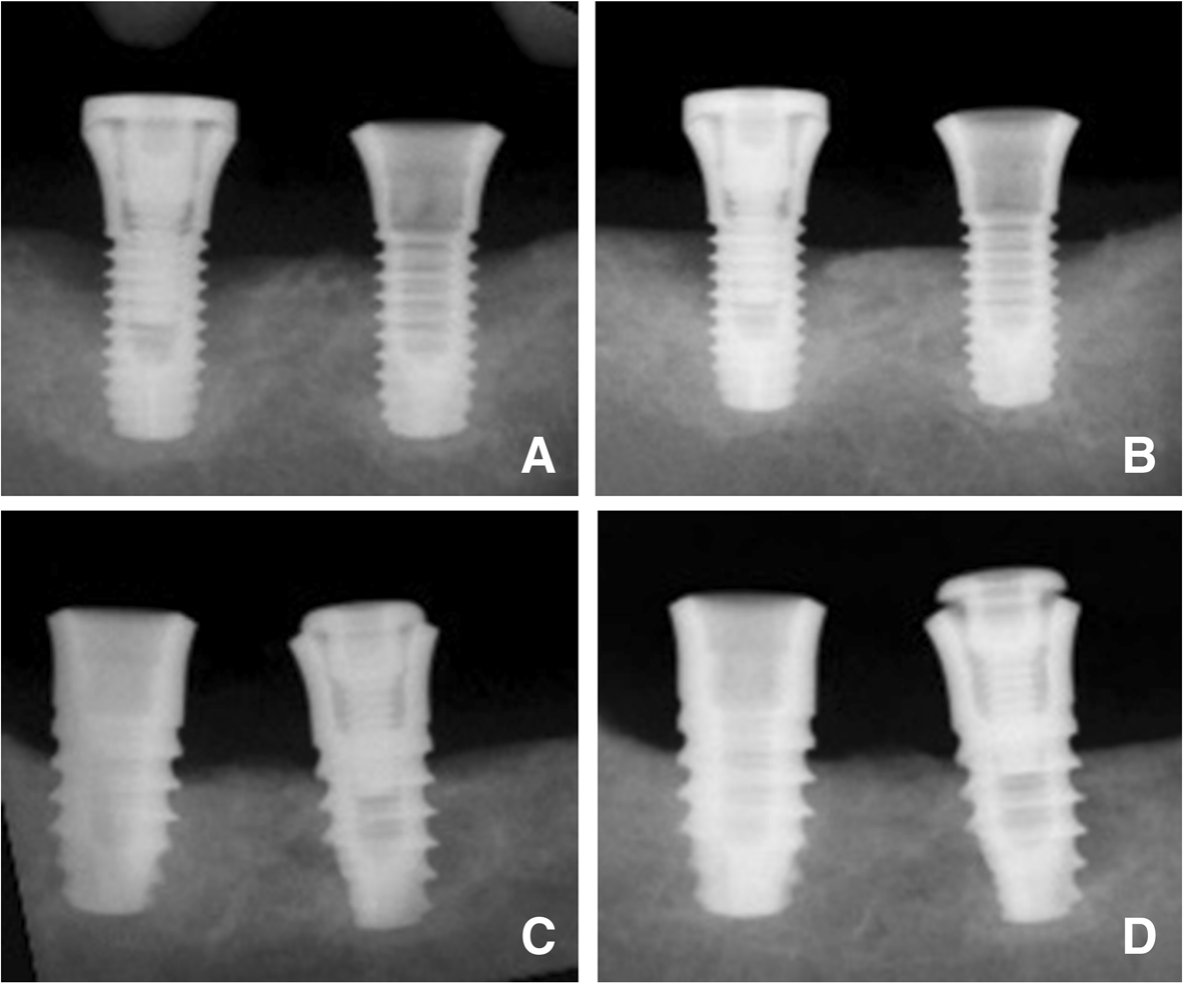
X-ray images. **a**, **b** Implants 3.3 mm in diameter (**a**) at the time of treatment and (**b**) after 5 months of healing. **c**, **d** Implants 4.25 mm (at the left) and 3.8 mm (at the right) in diameter (**c**) at the time of treatment and (**d**) after 5 months of healing

**Fig. 4:**
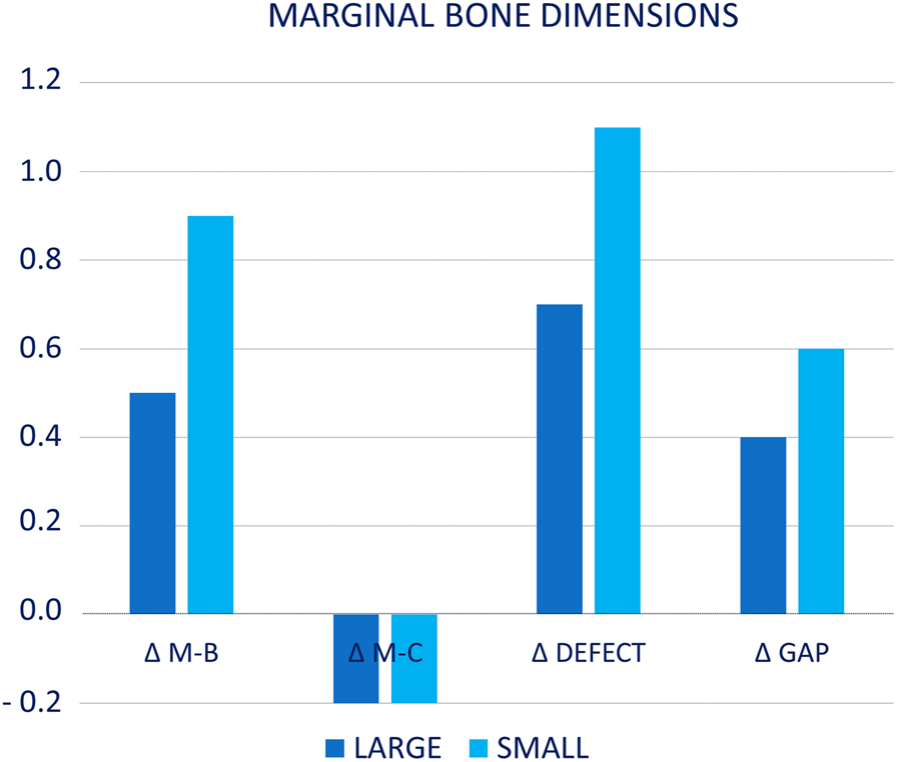
Graphs illustrating the radiographic change at the marginal bone 5 months after treatment. Mean gain of osseointegration and closure of the defect were observed in both groups

**Fig. 5:**
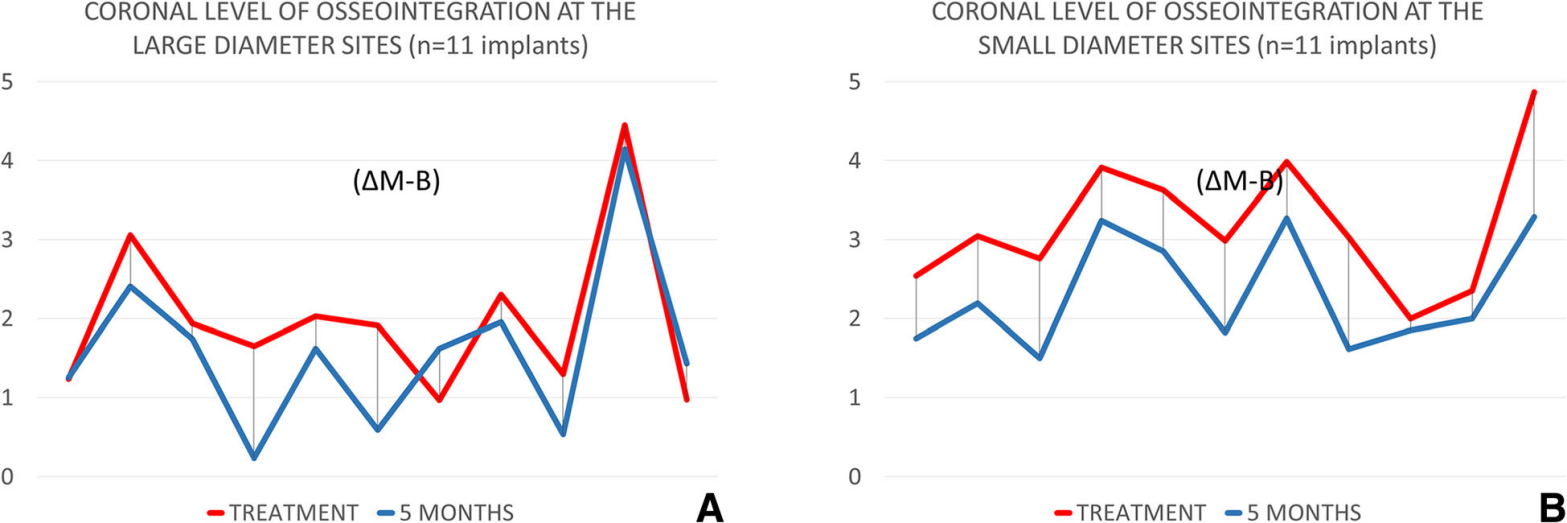
Changes of the coronal level of osseointegration (ΔM-B) evaluated for each implant. **a** Standard diameter group. **b** Narrow diameter group

At the radiographic evaluation, all sites presented marginal bone loss. A radiographic mean loss of marginal bone (M-B) at the mesio-distal aspects of 2.2 ± 1.0 mm at the standard implants, and of 3.2 ± 0.4 mm at the narrow implants were found (*p* = 0.116). Defects were present in both groups, being deeper and wider at the narrow compared to the standard implants (Table 1; Fig. 3). After 5 months of healing, a coronal gain in osseointegration (M-B) was observed in both groups, being 0.5 ± 0.6 mm at the standard implants and 0.9 ± 0.4 mm at the narrow implants (*p* = 0.345). The marginal bone defects presented a substantial closure both vertically and horizontally, mainly associated with bone formation within the bone defect and, to a less extent, to the resorption of the bone crest. In fact, the bone crest (M-C) presented a limited apical resorption after healing (− 0.2 mm) in both groups (Table 1; Fig. 4). Most of the implants presented a marginal bone gain (Fig. 5).

**Table 1:** Radiographic evaluation at the mesio-distal aspects. Mean values ± standard deviations and medians (25%;75% percentiles) of the two standard implants (3.8 mm and 4.25 mm) and of the two respective narrow implants (3.3 mm and 3.3 mm) at the time of treatment and after 5 months of healing. Differences (Δ) between periods and *p* values (*p* < 0.05) are also reported. *n* = 6

Data in millimeter	Standard				Narrow			
	M-B	M-C	V-defect	H-GAP	M-B	M-C	V-defect	H-GAP
Treatment	2.2 ± 1.0 1.8 (1.5;3.0)	1.2 ± 1.01.2 (0.8;2.1)	0.9 ± 0.60.8 (0.5;1.3)	0.5 ± 0.40.5 (0.2;0.7)	3.2 ± 0.43.0 (3.0;3.2)	1.6 ± 0.61.7 (1.1;2.1)	1.5 ± 0.61.9 (1.2;1.9)	0.8 ± 0.40.7 (0.5;1.0)
5 months after treatment	1.7 ± 0–9 1.7 (1.2;2.3)	1.4 ± 1.01.5 (0.5;2.2)	0.3 ± 0.3 0.1 (0.1;0.3)	0.1 ± 0.10.0 (0.0;0.1)	2.3 ± 0.42.5 (2.3;2.5)	1.8 ± 0.41.8 (1.6;2.1)	0.5 ± 0.60.4 (0.0;0.8)	0.2 ± 0.20.1 (0.0;0.4)
p standard vs narrow at treatment	0.116	0.463	0.249	0.249				
p treatment vs. 5 months after treatment	0.173	0.249	0.046	0.043	0.028	0.249	0.028	0.028
Δ treatment and 5 months after treatment	0.5 ± 0.6 0.4 (0.3;0.9)	− 0.2 ± 0.4–0.3 (− 0.4;− 0.1)	0.7 ± 0.5 0.7 (0.5;0.9)	0.4 ± 0.4 0.4 (0.2;0.5)	0.9 ± 0.4 0.8 (0.6;1.2)	− 0.2 ± 0.3–0.2 (− 0.4;0.1)	1.1 ± 0.5 1.1 (0.7;1.2)	0.6 ± 0.4 0.5 (0.5;0.7)
p standard vs. narrow	0.249	0.917	0.599	0.600				

Δ = difference; *M* = coronal margin of the rough surface; *B* = most coronal contact of the bone to the implant surface; *C* = top of the marginal bone crest; *V-defect* = vertical defect; *H-GAP* = horizontal defect

**Fig. 6:**
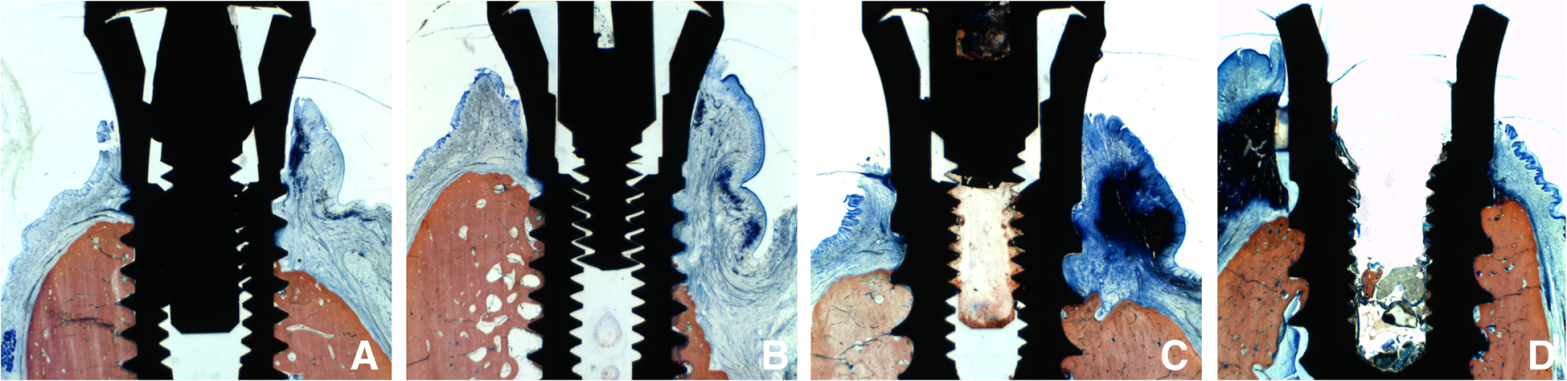
Microphotographs of ground section representing the healing at (**a**, **b**) 3.3 mm implants, and (**c**) 3.8 mm and (**d**) 4.25 mm implants

At the histological analyses (Table 2; Fig. 6), small amounts of inflammatory infiltrate were seen in both groups, with very few osteoclasts close to the marginal bone around implants. At the standard implants, the coronal level of osseointegration and the bone crest were located at 2.1 ± 0.8 mm and 2.0 ± 0.7 mm, respectively. The corresponding levels at the narrow implants were 2.9 ± 0.5 mm (*p* = 0.075 between groups) and 2.3 ± 0.3 mm (*p* = 0.400 between groups) for the standard and narrow implants, respectively.

**Table 2:** Histological evaluation in millimeters at buccal-lingual aspects after 5 months of healing. Mean values ± standard deviation and median (25%;75% percentiles) of the two regular implants (3.8 mm and 4.25 mm) and of the two respective narrow implants (3.3 mm and 3.3 mm). *p* values (*p* < 0.05); *n* = 6

Data in millimeter	Standard		Narrow	
	M-B	M-C	M-B	M-C
5 months of healing	2.1 ± 0.8 2.1 (1.4;2.5)	2.0 ± 0.7 2.1 (1.4;2.5)	2.8 ± 0.3 2.8 (2.7;2.9)	2.3 ± 0.3 2.3 (2.2;2.4)
p standard vs. narrow	0.116	0.400		

*M* = coronal margin of the rough surface; *B* = most coronal contact of the bone to the implant surface; *C* = top of the marginal bone crest

## Discussion

In the present experiment, a peri-implantitis was induced maintaining submucosal ligatures for 3 months around implants with different diameters. Plaque control was discontinued during these 3 months, and for one more month after ligatures removal. A higher loss of marginal bone of 1 mm and deeper marginal vertical defects were observed at the narrow compared to the standard implants, even though no statistically significant differences were found. After 5 months from the surgical treatment of the lesions, a bone gain was radiographically observed both at the standard and narrow implants.

Comparisons in the clinical outcomes between narrow and standard implants have been performed and no differences were found in the marginal bone level, implant survival, success rate, and clinical performances [13, 14]. However, the present study showed a higher progression of peri-implantitis at the narrow compared to the standard implants. It might be speculated that the larger volumes of soft and hard tissues surrounding standard implants compared to narrow implants may require a longer time to induce similar amount of tissue destruction.

A higher gain of the coronal level of the osseointegration was obtained at the narrow implants (0.9 mm) compared to the standard implants (0.5 mm), even though the difference was not statistically significant (*p* = 0.249). This outcome might be related to the presence of deeper intraosseous defects around the narrow implants (1.5 mm) compared to the standard implants (0.9 mm), condition that allowed the formation of 4-walls infrabony defects to a larger extent at the standard compared to the narrow implants. This might have influenced the amount of new bone formed within the marginal defect [18–21]. The marginal bone defects were found almost resolved mainly by new bone formation, while bone crest resorption scarcely influenced the outcome, being about 0.2 mm in both groups. It has to be considered that the moderate roughness of the surface used in the present study may have contributed to the closure of the defect and to the improved levels of the marginal bone, confirming the results from experimental studies in dogs [22–25].

A surgical approach has been shown to be more effective compared to a non-surgical treatment [26, 27]. The surface of the implants was decontaminated with gazes soaked with saline and irrigation with saline, treatment repeated several times to improve the cleaning effect. This method of decontamination was already described previously in various studies [9–12]. Nevertheless, it has been reported that there is no sufficient evidence to recommend a specific treatment for peri-implantitis [28], even though the use of air-abrasive devices seems to produce an improved surface decontamination compared to other mechanical approaches [29].

In an experiment in dogs, two different methods of decontamination were compared [16]. Implants were installed in the premolar region of the mandible of dogs. After 3 months, ligatures were placed for 3 months to induce peri-implantitis and plaque accumulation was allowed. The ligatures were removed and the plaque was left to accumulate for a further month. Subsequently, a corrective surgical debridement of the infected sites was performed. The surface was decontaminated using either a rotatory titanium brush or, similarly to the present study, gauzes soaked with saline followed by irrigation with saline. Both methods yielded a marginal bone gain and a low concentration of inflammatory cells.

It has been shown that the turned surfaces present a lower progression of peri-implantitis [9] and better outcomes after treatment compared to the rough surfaces [7, 8]. Nevertheless, a systematic review on experimental studies regarding the treatment of peri-implantitis reported better results in marginal bone gain at rough surfaces compared to the standard turned surfaces [30]. Also in the present study, the implants used had a modified rough surface and a gain of 0.5–0.9 mm was obtained after surgical treatment and plaque control for 5 months.

The histological data revealed a stable situation in most of the cases with low amounts of inflammatory infiltrates. A more apical location of both the coronal level of osseointegration and of the bone crest at the narrow compared to the standard implants was found. This is obviously related to the higher bone resorption that occurred to the narrow implants compared to the standard implants during the phase of peri-implantitis induction.

The present study presents various limitations, such as the model used, composed of young and healthy dogs that did not present any signs of periodontitis at the time of extractions. Moreover, the peri-implantitis was not spontaneous, but instead induced in a short time using ligature. Conversely, an established disease in human takes generally several years, and the first signs of infection may be seen in the majority of cases after 3 years of function [31]. The small sample and the reduced difference in the diameter of the implants of the two groups represent a further limitation that did not allow to reach statistically significant differences in marginal bone loss after peri-implantitis induction and bone gain after surgical treatment. Studies using a larger number of animals and groups of implants with a larger difference in diameter may offer more chances to reach a statistically significant difference.

Due to the limitations illustrated above, the present study only disclosed a tendency of a faster progression (+ 1 mm) of the peri-implantitis at narrow compared to standard implants, without reaching a statistically significant difference in marginal bone loss.

This, in turn, means that the hypothesis of the present study that the diameter of the implants might influence the progression of the disease and outcome of a corrective surgical debridement was rejected.

In conclusion, the narrow implants showed a tendency of a faster progression of the induced peri-implantitis compared to standard implants. The implant diameter did not influence the outcome of a surgical treatment of an induced peri-implantitis.

## Abbreviation

B: Most coronal level of osseointegration
C: Top of the bone crest
H-GAP: Distance between the implant surface and the bone crest
IS: Implant shoulder
M: Coronal margin of the rough surface
V-defect: Vertical defect
Δ: Difference
